# Assessing the progress in implementing population-based policies to reduce the burden of noncommunicable diseases in Eastern Europe and Central Asia, 2010–2024

**DOI:** 10.1093/heapol/czag055

**Published:** 2026-04-16

**Authors:** Anastasiya Dumcheva, Tiina Laatikainen, Ivo Rakovac, Jaakko Nevalainen, Pekka Nuorti

**Affiliations:** Health Sciences Unit, Faculty of Social Sciences, Tampere University, Arvo Ylpön katu 34, Tampere 33014, Finland; Institute of Public Health and Clinical Nutrition, University of Eastern Finland, Yliopistonranta 8, Kuopio 70210, Finland; Unit Head NCD Surveillance, Special Initiative on Noncommunicable Diseases and Innovation, WHO Regional Office for Europe, UN City, Marmorvej 51, DK-2100 Copenhagen, Denmark; Health Sciences Unit, Faculty of Social Sciences, Tampere University, Arvo Ylpön katu 34, Tampere 33014, Finland; Health Sciences Unit, Faculty of Social Sciences, Tampere University, Arvo Ylpön katu 34, Tampere 33014, Finland

**Keywords:** noncommunicable diseases, policies, tobacco, alcohol, nutrition, physical activity, Eastern Europe, Central Asia, best buys

## Abstract

Premature mortality from noncommunicable diseases (NCDs) remains high in twelve Eastern Europe and Central Asia (EECA) countries—Armenia, Azerbaijan, Belarus, Georgia, Kazakhstan, Kyrgyzstan, Moldova, Russia, Tajikistan, Turkmenistan, Ukraine, and Uzbekistan. Although WHO-recommended ‘Best Buys’ offer effective strategies to reduce NCDs, their implementation in EECA remains poorly documented. We conducted a cross-country, retrospective analysis of the adoption and implementation of population-level NCD ‘Best Buys’ interventions targeting tobacco, alcohol, diet, and physical activity across EECA from 2010 (or earliest available year) to 2024 (or latest available year), aiming to identify progress, gaps, and priorities for action. Data were sourced from WHO NCD Country Capacity Surveys and other global databases and monitoring reports. A scoring system (0–1) captured implementation status, and spider charts and summary tables visualized trends over time. Tobacco control showed the most progress, with widespread adoption of taxation and graphic warnings. However, the implementation of smoke-free laws, cessation support, and media campaigns was inconsistent. Alcohol policies varied: most countries increased taxes and banned advertising, but gaps persisted in sales restrictions, health warnings, and treatment services. Adoption of nutrition policies remained inconsistent, with substantial gaps in food reformulation, labelling, fiscal tools, and education. Physical activity campaigns were common, but integration into healthcare systems was poorly documented. Disparities in implementation were observed across and within countries, in terms of the number and combination of ‘Best Buys’ strategies adopted. Despite some progress, major gaps remain in the implementation of population-level NCD ‘Best Buys’ across EECA. Greater prioritization of cost-effective tobacco, alcohol, nutrition, and physical activity strategies is needed. Subregional and country-level analyses of NCD ‘Best Buys’ implementation over time can help policymakers identify progress and gaps, guiding targeted, evidence-informed action to address shared behavioural risks and thereby prevent many NCDs and contribute to equitable and sustainable health outcomes.

Key messages

- Noncommunicable diseases (NCDs) are the leading cause of premature mortality in Eastern Europe and Central Asia (EECA)—including Armenia, Azerbaijan, Belarus, Georgia, Kazakhstan, Kyrgyzstan, Moldova, Russia, Tajikistan, Turkmenistan, Ukraine, and Uzbekistan—and contribute substantially to health and economic burdens.
- Although many EECA countries have committed to reducing NCDs, the adoption and implementation of the WHO NCD ‘Best Buys’—a set of effective, evidence-based interventions—remain poorly documented.
- This study provides the first comprehensive cross-country assessment of population-level NCD ‘Best Buys’ on tobacco and alcohol control, healthy nutrition and physical activity across 12 EECA countries from 2010 to 2024 in order to identify progress, gaps, and priorities for multisectoral action.
- Findings indicate that policymakers should prioritize stronger tobacco and alcohol control through taxation, advertising bans, and enforcement, and invest more in nutrition and physical activity interventions; subregional monitoring of ‘Best Buys’ can further support tailored, evidence-informed policies and accelerate progress towards NCD reduction goals.

## Introduction

### NCDs as a global issue

Noncommunicable diseases (NCDs)—including cardiovascular diseases, cancer, diabetes, and chronic respiratory diseases—are the leading cause of death globally, responsible for over 70% of all deaths (Institute for Health Metrics and Evaluation 2023, WHO 2025). They also impose a significant financial burden on health systems and individuals, particularly in low- and middle-income countries (WHO 2025). Major risk factors such as tobacco use, unhealthy diets, physical inactivity, alcohol consumption, and air pollution contribute to this growing epidemic (Institute for Health Metrics and Evaluation 2023, WHO 2025). NCDs may significantly increase healthcare spending and can drive households into poverty due to high out-of-pocket expenses (Muka et al. 2015).

### The role of NCD policies

NCD policies are essential in reducing the disease burden by addressing key risk factors through prevention, early detection, and treatment strategies (WHO 2013a). Effective policies can promote healthier environments (WHO 2019a, 2023a), ensure access to quality healthcare (Koohpayezadeh et al. 2021), and implement health education campaigns (Allen et al. 2017, Dodd et al. 2022). Comprehensive approaches—such as legislation and taxation on harmful products (e.g. tobacco, alcohol, and sugary drinks) (Wagenaar et al. 2009, Ekpu and Brown 2015, Golden et al. 2016, Erkoyun et al. 2017, Irwin et al. 2017, Teng et al. 2019, Hiscock et al. 2021, Andreyeva et al. 2022, Guindon et al. 2022), food reformulation, and regulation of food labelling (Mazzocchi et al. 2015, Hashem et al. 2019, Gressier et al. 2021, Kelly et al. 2024, WHO 2026a)—have proven effective in reducing NCD-related morbidity and mortality. However, corporate influence and lobbying can hinder the implementation of such policies, particularly those targeting unhealthy commodities (Allen et al. 2021, 2023).

### Remaining challenges

Despite some progress, the implementation of policies targeting tobacco, alcohol, and other NCD risk factors remains inconsistent. While tobacco use has generally declined, alcohol consumption continues to pose challenges in some regions (Allen et al. 2021, 2023). Rising rates of overweight and obesity underscore the need for policies promoting healthier diets and physical activity (WHO 2004, World Obesity 2024). Additionally, high blood pressure remains the leading risk factor for premature mortality (Our World in Data 2021).

Many countries are not on track to meet the Sustainable Development Goals (SDG) target for reducing premature NCD mortality. Progress has been particularly slow in low- and middle-income countries (LMICs) (WHO 2025). According to the World Health Organization (WHO), only 14 countries were on track in 2022 to meet the SDG 3.4.1 target—a one-third relative reduction in premature mortality from four major NCDs among people aged 30–69 by 2030, compared with 2015 levels. The WHO emphasized that ‘progress in reducing premature deaths from NCDs is insufficient’ in most parts of the world, especially in LMICs (WHO 2022a). The latest Lancet NCD Countdown 2030 reports from 2020 and 2022 similarly concluded that most LMICs are ‘off track’ to achieve this target, warning that without urgent action, over 85% of these countries will miss SDG 3.4.1 (Bennett et al. 2018, 2020, Nishtar et al. 2018, The Lancet 2018, 2020, 2022, Watkins et al. 2022).

In the WHO European Region, the Eastern European and Central Asian (EECA) subregion—which comprises former Soviet Union countries that did not join the European Union—has shown particularly slow progress towards achieving SDG 3.4.1 (Dumcheva et al. 2024). This subregion includes Armenia, Azerbaijan, Belarus, Georgia, Kazakhstan, Kyrgyzstan, Moldova, Russia, Tajikistan, Turkmenistan, Ukraine, and Uzbekistan. If current trends in NCD mortality persist, it is estimated that it could take approximately 50 years for these countries to reach the levels of premature NCD mortality of the European Union member states that joined before May 2004 (Jakab et al. 2018).

### WHO NCD ‘best buys’ and ‘quick buys’ strategies

WHO has identified a set of the most effective population- and individual-level, intersectoral interventions to address the NCD burden, known as ‘Best buys and other recommended interventions for the prevention and control of noncommunicable diseases’ (WHO 2024a). These include evidence-based population measures targeting tobacco and alcohol consumption, unhealthy diets, and physical inactivity, as well as individual-based interventions for the prevention, early detection, and treatment of cardiovascular diseases, cancers, chronic respiratory diseases, and diabetes. Of the 90 recommended interventions, 28 are classified as ‘Best Buys’ for which the WHO has identified an average cost-effectiveness ratio of ≤ 100 international dollars (I$) per healthy life year (HLY) gained. A second group comprises effective interventions with cost-effectiveness ratios > I$100 per HLY gained, which still represent good value for money depending on national thresholds. These categories reflect the potential of the interventions to deliver substantial health gains for relatively modest investment, positioning them as priority actions for NCD prevention and control. A third category comprises recommended interventions supported by evidence of effectiveness but lacking available cost-effectiveness analyses. The first list of NCD Best Buys and other recommended interventions was released by WHO in 2017 (WHO 2017a), and most population-level measures to reduce tobacco and alcohol use, improve diet, and increase physical activity remain unchanged in the 2024 update.

More recently, WHO also introduced ‘Quick Buys’, a prioritized subset of the Best and other recommended population-level and individual-level interventions that can deliver measurable improvements in population health within 5 years or less, and in some cases almost immediately (Galea et al. 2025). Together, these frameworks provide a structured approach for assessing national policy implementation and identifying gaps in the adoption of high-impact NCD interventions. However, a comprehensive assessment of NCD policy implementation based on the latest WHO recommendations has not yet been conducted in EECA subregion.

We assessed the progress of population-based NCD ‘Best Buys’ policy implementation in 12 EECA countries from 2010 (or the earliest available year) to 2024 (or the latest available year) to document trends, identify gaps and identify priority actions for NCD policy implementation.

## Materials and methods

We used the updated list of WHO Best Buys (WHO 2024a) as a framework to analyse the adoption and implementation status of NCD policies across 12 EECA countries, covering the period from 2010 (or the earliest available year) to 2024 (or the most recent available year). The analysis focused on population-based interventions targeting major NCD risk factors: tobacco use, alcohol consumption, unhealthy diet, and physical inactivity.

The primary data source was the NCD Country Capacity Surveys (WHO 2026b) conducted in 2010, 2013, 2015, 2017, 2019, 2021, and 2023 by WHO Regional Office for Europe. These surveys are implemented at regular intervals and collect detailed information on national NCD policies, governance structures, surveillance systems, and population-level interventions using standardized questionnaires and harmonized data-collection procedures across WHO Member States. Their standardized design enables reliable comparison across countries and over time, providing a consistent basis for assessing policy implementation progress.

These were supplemented with a range of publicly available sources including the WHO Global Health Observatory (World Health Organization 2025), WHO Reports on the global tobacco epidemic (WHO 2013b, 2015a, 2017b, 2019b, 2021a, 2021b, 2023b), WHO Global Status Reports on Alcohol and Health (WHO 2011, 2014, 2018), WHO Progress Monitor Reports (WHO 2015b, 2017c, 2020, 2022a), the NCD Document Repository (WHO 2026c), and the Status Reports on Marketing of Breast-Milk Substitutes (WHO et al. 2016, 2018, 2020, 2022, 2024). These sources compile information through standardized WHO questionnaires completed by national focal points, routine reporting mechanisms, and systematic reviews of national legislation and policy documents. All submissions undergo WHO verification procedures, including internal consistency checks, cross-referencing with official legal texts, and follow-up with country authorities. Together, these harmonized data collections provide comparable, validated information on tobacco control, alcohol policy, nutrition-related regulation, and broader NCD policy commitments across countries and over time. The description of which data source was used for each policy assessment is presented in Supplementary Appendix A.

After compiling and validating all data sources, we systematically extracted information on the presence and/or implementation status of policies using a structured extraction template that included the name of the WHO NCD Best Buys intervention, operational definitions of each policy assessed, the earliest and latest data points available for each policy, and the corresponding data sources (Supplementary Appendix A). When multiple data sources were available for the same policy, the research team selected the source that provided the greatest level of detail and the longest temporal coverage.

Data extraction was conducted by one researcher, followed by review and discussion within the research team to verify accuracy, resolve discrepancies, and ensure consistency across countries and policy domains. This verification process strengthened the reliability and reproducibility of the extracted dataset.

To assess the degree of policy implementation, we applied a scoring system: a score of 1 indicated full adoption or implementation, 0 indicated non-implementation or unavailable data, and intermediate values (e.g. 0.5) reflected partial implementation. Detailed scoring criteria, definitions, data sources, and descriptions for each policy are provided in Supplementary Appendix A.

To compare countries and track progress over time, we visualized policy implementation using spider charts at two points: the earliest and the latest available years for each country. If data for a specific policy were available for only one of these time points, we conservatively assumed non-implementation at the missing time point and assigned a score of 0. This approach ensured that the visual comparisons accurately reflected both policy action and data availability.

Visualizations were limited to NCD Best Buys for which cost-effectiveness analyses were available. Policies included in the WHO Best Buys but lacking such analyses were incorporated only when relevant implementation data were available. In these cases, key findings were summarized descriptively and presented in summary tables.

The data were summarized and analysed using R software, version 4.3.1 (The R Foundation 2025). Microsoft Copilot was used to support language editing, which the authors ensured accuracy and integrity through manual review.

## Results

### Tobacco policies

**Figure 1:**
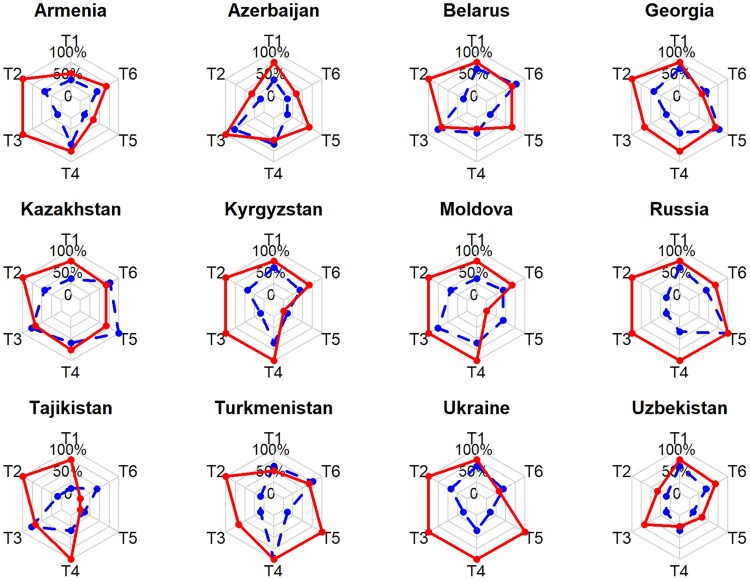
Progress in tobacco-control policy implementation in Eastern Europe and Central Asia: earliest (blue, dotted line) vs latest (red, solid line) assessment. Box file legend for Figure 1: List of tobacco policies assessed (earliest year, latest year): T1—increase excise taxes and prices on tobacco products (2008, 2022). T2—implement large graphic health warnings on all tobacco packages, accompanied by plain/standardized packaging (2007, 2022). T3—enact and enforce comprehensive bans on tobacco advertising, promotion, and sponsorship (2007, 2022). T4—eliminate exposure to second-hand tobacco smoke in all indoor workplaces, public places, and public transport (2007, 2022). T5—implement effective mass media campaigns that educate the public about the harms of smoking/tobacco use and second-hand smoke and encourage behaviour change (2010, 2022). T6—provide cost-covered, effective, population-wide support (including brief advice, national toll-free quit line services and mCessation) for tobacco cessation to all tobacco users and provide cost-covered effective pharmacological interventions to all tobacco users who want to quit, through the use of nicotine replacement therapy, bupropion, and varenicline (2007, 2022).

The study revealed notable progress in tobacco-control policies across EECA countries, alongside persistent disparities in implementation (Fig. 1). Detailed scoring criteria, definitions, and guidance on interpreting tobacco policy scores are provided in Supplementary Appendix A, and the assigned values used to construct Fig. 1 are presented in Supplementary Appendix B.

While all countries increased tobacco taxes from 2008 to 2022, none achieved a total tax share exceeding 75% of the retail price. Armenia and Turkmenistan reached ∼50% and therefore did not meet the WHO-recommended benchmark of at least 75% of the retail price.

Substantial progress was observed in the adoption of large graphic health warnings on tobacco packaging from 2007 to 2022, with the except for Azerbaijan and Uzbekistan. Half of the countries implemented comprehensive bans on direct and indirect forms of tobacco advertising, promotion, and sponsorship during this period. The remaining countries—Belarus, Georgia, Kazakhstan, Tajikistan, Turkmenistan, and Uzbekistan—enacted only partial bans.

Similarly, from 2007 to 2022, only six countries (Kyrgyzstan, Moldova, Russia, Tajikistan, Turkmenistan, and Ukraine) adopted comprehensive smoke-free legislation covering all indoor workplaces, public places, and public transport. The others implemented such legislation with varying degrees of restrictions.

The implementation of anti-tobacco mass media campaigns was inconsistent between 2010 and 2022. Russia maintained consistent efforts, while Turkmenistan and Ukraine showed notable improvements. Although most countries improved the availability of cost-covered, population-wide tobacco cessation support and services between 2007 and 2022, accessibility remained limited. Notably, Tajikistan experienced a decline in cessation service availability, and no country offered fully accessible, cost-covered national tobacco quitlines or nicotine replacement therapies.

Across countries, fiscal and regulatory measures, such as taxation and large graphic health warnings, were the most consistently implemented. In contrast, comprehensive advertising bans and smoke-free legislation were achieved in only about half of the countries, while anti-tobacco mass media campaigns and service-based cessation interventions showed the weakest progress. No EECA country has fully implemented all six recommended tobacco-control policies. Only Russia and Ukraine achieved full implementation of four policies. Kyrgyzstan, Moldova and Turkmenistan fully implemented three, Armenia and Tajikistan implemented two. Azerbaijan, Belarus, Georgia, and Kazakhstan each fully implemented only one tobacco-control policy, while Uzbekistan implemented none highlighting significant gaps in tobacco control across the region.

## Alcohol policies

**Figure 2:**
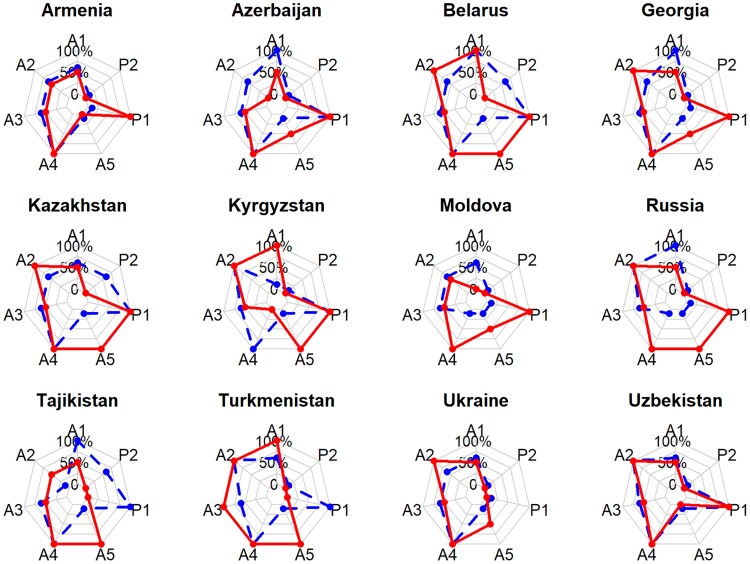
Progress in alcohol- and physical-activity policy implementation in Eastern Europe and Central Asia: earliest (blue, dotted line) vs latest (red, solid line) assessment. Box file legend for Figure 2: List of alcohol and physical activity policies assessed (earliest year, latest year): **A1**—increase excise taxes on alcoholic beverages (2015, 2022). **A2**—enact and enforce bans or comprehensive restrictions on exposure to alcohol advertising (across multiple types of media) (2015, 2022). **A3**—enact and enforce restrictions on the physical availability of retailed alcohol (via reduced hours of sale) (2015, 2022). **A4**—enact and enforce drink-driving laws and blood alcohol concentration limits via sobriety checkpoints (2014, 2018). **A5**—provide brief psychosocial intervention for persons with hazardous and harmful alcohol use (−, 2024). **P1**—implement sustained, population-wide, best-practice communication campaigns to promote physical activity, with links to community-based programmes and environmental improvements to enable and support behaviour change (2015, 2023). **P2**—provide physical activity assessment, counselling, and behaviour change support as part of routine primary health care services through the use of a brief intervention (2010, -).

The implementation of alcohol-related policies varied substantially across EECA countries. While some countries made notable progress in adopting evidence-based measures to reduce alcohol consumption, others showed limited or inconsistent progress (Fig. 2). Detailed scoring criteria, definitions, and guidance on interpreting alcohol policy scores are provided in Supplementary Appendix A, and the assigned values used to construct Fig. 2 are presented in Supplementary Appendix B.

From 2015 to 2022, most countries increased excise taxes on alcohol; however, full implementation was achieved only in Belarus, Kyrgyzstan, and Turkmenistan (WHO 2015b, 2017c, 2020, 2022a).

During the same period, countries made progress in banning alcohol advertising across various media platforms. By 2022, Armenia, Moldova, and Tajikistan had partially implemented such bans. Belarus, Georgia, Kazakhstan, and Ukraine transitioned from partial to full bans, while Kyrgyzstan, Russia, Turkmenistan, and Uzbekistan maintained existing full prohibitions (WHO 2015b, 2017c, 2020, 2022a).

Restricting alcohol sales hours is an effective strategy, yet from 2015 to 2022, only Turkmenistan fully implemented such restrictions. In other countries, alcohol remained widely accessible (World Health Organization 2025).

Most countries maintained national legal limits over years 2014–2018 for blood alcohol concentration while driving, ranging from ‘zero tolerance’ (e.g. Azerbaijan, Kazakhstan, Uzbekistan) to a maximum of 0.08% in Armenia (World Health Organization 2025). Data from Kyrgyzstan were unavailable for 2018, preventing assessment.

As of 2024, half of the EECA countries had implemented policies ensuring access to screening and brief intervention programmes for alcohol use (WHO 2024b). These programmes were available in Belarus, Kazakhstan, Kyrgyzstan, Russia, Tajikistan, and Turkmenistan. Azerbaijan, Georgia, Moldova, and Ukraine offered limited availability, while Armenia lacked these programmes entirely. Data for Uzbekistan were unavailable. Due to limited historical data, trends over time could not be assessed.

Across countries, regulatory measures, such as taxation, advertising bans, and drink-driving laws, were more consistently implemented than service-based interventions. In contrast, restrictions on alcohol availability and access to brief psychosocial support showed the weakest progress.

Turkmenistan was the only country to fully implement all five recommended cost-effective alcohol control policies. Belarus implemented four; Kazakhstan, Kyrgyzstan, and Russia implemented three. Georgia, Tajikistan, Ukraine and Uzbekistan fully implemented two, and Armenia, Azerbaijan and Moldova only one.

We also assessed additional alcohol-related Best Buys policies without cost-effectiveness evidence. The minimum legal age for purchasing alcohol was 18 years in most EECA countries, except in Uzbekistan (20 years), and in Kazakhstan and Turkmenistan (21 years) (World Health Organization 2025).

The Service Capacity Index for Alcohol Use Disorders reported by WHO as a composite measure of the strength of national treatment systems varied substantially across the 12 countries (Table 1). In interpreting these data, we followed WHO’s presentation of the index, in which higher values reflect more developed service capacity. For descriptive purposes, we considered values ≥0.5 to indicate relatively stronger system capacity and values <0.5 to reflect more limited or partial development of treatment services. Using this approach, Belarus, Kazakhstan, Moldova, Tajikistan, and Ukraine demonstrated comparatively stronger service capacity, each with an index value of 0.5 of higher. Armenia, Azerbaijan, Georgia, Kyrgyzstan, and Russia scored below 0.5, indicating more limited availability or organization of treatment services for alcohol use disorders. No data were available for Turkmenistan and Uzbekistan in the 2024 WHO report, preventing assessment of their service capacity (WHO 2024b).

**Table 1:** Service capacity index for alcohol use disorders.

Country	Index	Confidence interval	
		Lower limit	Upper limit
Armenia	0.22	0.15	0.3
Azerbaijan	0.48	0.39	0.57
Belarus	0.54	0.45	0.63
Georgia	0.15	0.09	0.21
Kazakhstan	0.51	0.42	0.6
Kyrgyzstan	0.22	0.14	0.29
Moldova	0.66	0.58	0.75
Russia	0.49	0.4	0.58
Tajikistan	0.76	0.68	0.83
Turkmenistan	No data	No data	No data
Ukraine	0.5	0.42	0.59

Source: WHO Global status report on alcohol and health and treatment of substance use disorders, 2024.

We also assessed the presence of health warnings on alcohol-related harm. This varied widely across the EECA subregion. Warnings about pregnancy and underage drinking appeared in both advertisements and in containers in several countries, while others lacked them entirely. Drink-driving warnings were more common in advertisements than on containers. Cancer warnings were rare, with only Georgia including them in advertisements. Most countries provided general risk information and alcohol content on containers, but labels indicating the number of standard alcoholic drinks were uncommon. Data on health warning labels for Tajikistan and Uzbekistan, were unavailable. For Uzbekistan, information was also missing for consumer information displayed on containers, the number of standard alcoholic drinks indicated, and alcohol content labelling (Table 2) (WHO 2024b).

**Table 2:** Provision of consumers with information about the content of alcoholic beverages and related harms.

Country	Health warning labels regarding								Consumer information displayed on containers	Number of standard alcoholic drinks displayed on container	Alcohol content displayed on containers
	Pregnancy		Underage drinking		Drink-driving		Cancer				
	Advertisements	Containers	Advertisements	Containers	Advertisements	Containers	Advertisements	Containers			
Armenia	No	Yes	No	Yes	No	No	No	No	Yes	No	Yes
Azerbaijan	No	No	No	No	No	No	No	No	Yes	No	Yes
Belarus	Yes	No	Yes	No	No	No	No	No	Yes	No	Yes
Georgia	Yes	Yes	Yes	Yes	Yes	Yes	Yes	No	Yes	No	Yes
Kazakhstan	Yes	No	Yes	No	Yes	No	No	No	Yes	No	Yes
Kyrgyzstan	No	No	No	No	No	No	No	No	No	No	No
Moldova	Yes	Yes	Yes	Yes	Yes	Yes	No	No	No	No	Yes
Russia	Yes	No	Yes	No	No	No	No	No	Yes	Yes	Yes
Tajikistan	No data								No	Yes	Yes
Turkmenistan	No	No	No	No	No	No	No	No	Yes	No	Yes
Ukraine	Yes	Yes	Yes	Yes	Yes	Yes	No	No	Yes	No	Yes
Uzbekistan	No data										

Source: WHO Global status report on alcohol and health and treatment of substance use disorders, 2024.

### Physical activity policies

Figure 2 includes two WHO Best Buys policies related to physical activity: (1) communication campaigns to promote physical activity and (2) integration of physical activity promotion into primary health care services. Detailed scoring criteria, definitions, and guidance on interpreting the policy scores for physical activity are provided in Supplementary Appendix A, and the assigned values used to construct Fig. 2 are presented in Supplementary Appendix B.

From 2015 to 2023, most EECA countries implemented communication campaigns to promote physical activity, recognizing their importance in preventing noncommunicable diseases. However, Tajikistan, Turkmenistan, and Ukraine did not implement such campaigns in a sustained manner, potentially limiting their long-term impact.

Assessing the integration of physical activity promotion into primary health care services proved challenging due to limited data availability. The most recent data, from 2010, indicated that only Belarus, Kazakhstan, and Tajikistan reported having government-approved national guidelines or protocols for physical activity assessment, counselling, and behaviour change support within routine primary health care services. The absence of updated data suggests that these services remain underdeveloped, or that their implementation is not systematically monitored.

### Healthy nutrition policies

**Figure 3:**
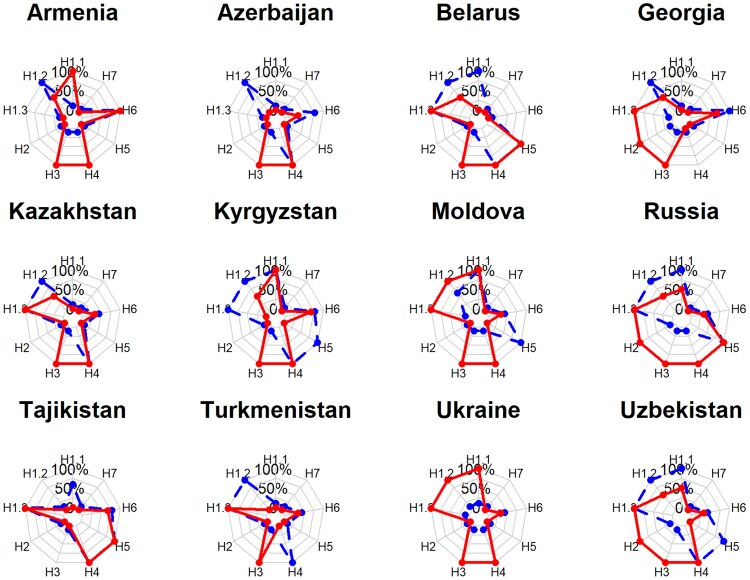
Progress in healthy nutrition policy implementation in Eastern Europe and Central Asia (EECA): earliest (blue, dotted line) vs latest (red, solid line) assessment. Box file legend for Figure 2: List of healthy nutrition policies assessed (earliest year, latest year): H1—Reformulation policies for healthier food and beverage products—assessed separately for **H1.1**—reduction of saturated fats (2015, 2023), **H1.2**—elimination of *trans*-fatty acids (2019, 2023), and **H1.3**—reduction of sodium (2015, 2023). **H2**—front-of-pack labelling as part of comprehensive nutrition labelling policies for facilitating consumers’ understanding and choice of food for healthy diets (2021, 2023). **H3**—public food procurement and service policies for healthy diets (−, 2023). **H4**—behaviour-change communication and mass-media campaigns for healthy diets (2015, 2023). **H5—**policies to protect children from the harmful impact of food marketing on diet (2015, 2023). **H6**—protection, promotion, and support of optimal breastfeeding practices (2016, 2024). **H7**—taxation on sugar-sweetened beverages as part of comprehensive fiscal policies to promote healthy diets (2015, 2023).

Progress in implementing healthy nutrition policies varied substantially across the EECA subregion (Fig. 3). Detailed scoring criteria, definitions, and guidance on interpreting the policy scores for healthy nutrition are provided in Supplementary Appendix A, and the assigned values used to construct Fig. 3 are presented in Supplementary Appendix B. Only a few countries strengthened government policies to reduce saturated fatty acids from 2015 to 2023. Armenia, Kyrgyzstan, Moldova, and Ukraine implemented mandatory reformulation measures, while Russia and Uzbekistan introduced such policies on a voluntary basis.

From 2019 to 2023, only Moldova and Ukraine adopted mandatory policies to eliminate *trans* fats in all food products, setting a limit of 2 g of industrially produced *trans*-fatty acids per 100 g of total fat in all foods. Other countries relied on voluntary measures.

From 2015 to 2023, several countries strengthened salt-reduction policies, including Georgia, Moldova, and Ukraine. Others—Belarus, Kazakhstan, Russia, Tajikistan, Turkmenistan, and Uzbekistan—maintained existing policies without further advancement. Armenia, Azerbaijan, and Kyrgyzstan did not implement salt-reduction policies.

Despite the importance of front-of-package labelling to help consumers make healthier choices, adoption of such policies identifying foods high in saturated fats, *trans* fats, free sugars, or salt was slow over 2021–2023. Only Georgia, Russia, and Uzbekistan implemented these measures.

We were unable to assess the implementation of public food procurement and service policies for healthy diets over time due to limited data availability. However, by 2023, all EECA countries except Tajikistan had implemented such policies to promote healthier diets.

From 2015 to 2023, most EECA countries actively implemented behaviour-change communication and mass-media campaigns to promote healthier diets. These activities expanded in Armenia, Moldova, Russia, and Ukraine, while Azerbaijan, Belarus, Kazakhstan, Kyrgyzstan, Tajikistan, and Uzbekistan maintained regular implementation. Georgia and Turkmenistan did not report undertaking such initiatives.

Despite growing concerns about the impact of unhealthy food marketing on children, most EECA countries—including Armenia, Azerbaijan, Georgia, Kazakhstan, Kyrgyzstan, Moldova, Turkmenistan, Ukraine, and Uzbekistan—did not strengthen policies regulating the marketing of foods and non-alcoholic beverages high in saturated fats, *trans* fats, free sugars, or salt over 2015–2023. By 2023, only Belarus, Russia and Tajikistan reported measures addressing cross-border marketing impacts on children.

Implementation of policies to protect, promote, and support breastfeeding practices from 2016 to 2024 also varied. Most countries incorporated some provisions of the International Code of Marketing of Breast-milk substitutes into their legislation, including Azerbaijan, Kazakhstan, Moldova, Russia, Turkmenistan, Ukraine, and Uzbekistan. Georgia, Kyrgyzstan, and Tajikistan had moderately aligned their policies with the Code, while Armenia achieved substantial alignment. Belarus did not introduce legal measures or provide relevant information (WHO et al. 2016, 2018, 2020, 2022, 2024).

Over 2015–2023, none of the EECA countries introduced taxation on sugar-sweetened beverages.

Across countries, reformulation policies targeting the reduction of saturated fats, elimination of *trans*-fatty acids, and reduction of sodium were most notably implemented in Ukraine and Moldova, which reported progress across all three domains. Other countries introduced reformulation measures with varying degrees of advancement or limited progress over time. Behaviour-change campaigns, and public food procurement, and service policies for healthy diets were more commonly implemented than front-of-pack labelling, policies to protect children from harmful food marketing, or measures to support optimal breastfeeding practices. Taxation policies on sugar-sweetened beverages were absent across all EECA countries.

Because reformulation policies were assessed across three separate components—reduction of saturated fats, elimination of *trans*-fatty acids, and reduction of sodium—these were counted as three distinct policy areas rather than a single composite measure, resulting in a total of nine nutrition policies assessed. No EECA country implemented all nine policies to their full potential. Moldova, Russia, and Ukraine demonstrated the strongest performance, each implementing five of the nine policies. Armenia, Belarus, and Uzbekistan followed with four implemented policies. Georgia, Kazakhstan, Kyrgyzstan, and Tajikistan implemented three, while Azerbaijan and Turkmenistan showed the lowest levels of progress, with only two policies implemented to their full potential.

We also assessed several other recommended Best Buys policies on healthy nutrition that lack cost-effectiveness evidence. Menu labelling was not introduced in any country. Subsidies for healthy foods and beverages (e.g. fruits and vegetables) as part of fiscal policies were also absent across the EECA subregion. Nutrition education and counselling in settings such as preschools, schools, workplaces and hospitals were difficult to assess due to limited data; the most recent data from 2010 showed that only Belarus, Russia, and Tajikistan had such programmes. No information was found on policies to limit portion and package sizes as a strategy to reduce excessive intake of calories, sugars, sodium, or unhealthy fats.

## Discussion

The study revealed notable progress in the implementation of WHO Best Buys policies targeting key NCD risk factors across Eastern Europe and Central Asia countries, though substantial gaps remain. Tobacco-control policies showed the most consistent improvement, particularly in taxation and graphic health warnings. However, the implementation of comprehensive smoke-free laws, cessation support, and media campaigns was uneven. Alcohol policies varied: while most countries increased taxes and banned advertising, gaps persisted in sales restrictions, health warnings, and treatment services. Nutrition policies remained inconsistently adopted, with major gaps in food reformulation, labelling, fiscal tools, and education. Physical activity campaigns were common, but integration into primary health care was poorly documented.

These findings align with previous research indicating positive trends in strengthening tobacco-control following the ratification of the Framework Convention on Tobacco Control (Global Alliance For Tobacco Control 2018), while also highlighting persistent disparities in policy implementation within the EECA subregion (WHO 2022a, 2023b, Ruokolainen et al. 2024). In particular, the EECA countries reflect the global pattern of slow progress in fully implementing smoke-free policies in indoor public places, workplaces, and public transport, as well as in raising tobacco taxes—despite these being among the most effective and rapidly impactful measures (WHO 2023b, Galea et al. 2025). The EECA subregion also mirrors global trends in limited adoption of anti-tobacco mass media campaigns and tobacco-cessation services, alongside stronger progress in implementing large graphic health warnings on packaging (WHO 2023b). However, while WHO reports widespread global advances in banning tobacco advertising, promotion, and sponsorship (WHO 2023b), only half of the EECA countries have implemented these policies comprehensively.

Importantly, none of the EECA countries have fully implemented all six recommended Best Buys tobacco-control policies. Only Russia and Ukraine achieved full implementation of four policies, whereas 5 of the 12 countries fully implemented only one policy or none. This suggests substantial gaps in tobacco policy uptake and underscores the need for renewed efforts to support comprehensive, evidence-based action across the EECA subregion.

In terms of alcohol control, the EECA subregion largely reflects the global trend described by WHO, where ‘most countries reported no progress on the Best Buys in alcohol policy since 2010’ (WHO 2022a, 2024b). Consistent with this, most EECA countries have demonstrated limited advancement. Notable progress was observed in Belarus, Kazakhstan, Kyrgyzstan, Russia, and Turkmenistan, where several alcohol-related policies were strengthened.

However, several characteristics of the EECA subregion stand out. While WHO reports that nearly all countries globally impose alcohol excise taxes (WHO 2024b), only Belarus, Kyrgyzstan, and Turkmenistan have fully implemented this highly effective measure (Galea et al. 2025). Additionally, although comprehensive bans on alcohol advertising and drink-driving laws (particularly those setting blood alcohol concentration limits below 0.05%) remain uncommon globally (WHO 2024b, World Health Organization 2025), the EECA countries have shown relatively stronger progress in adopting these specific measures.

Nonetheless, restricting the physical availability of alcohol—such as through reduced sales hours—remains a major challenge. To date, only Turkmenistan has fully implemented this measure. Furthermore, brief psychosocial interventions for individuals with hazardous or harmful alcohol use are available in only half of the EECA countries, indicating persistent gaps in service delivery.

Our findings align with previous research documenting uneven implementation of public education and awareness campaigns promoting physical activity (WHO 2022a, 2022b). In the EECA subregion, only half of the countries conducted such campaigns within the past 2 years, with notable gaps in Tajikistan, Turkmenistan, and Ukraine, where no sustained initiatives were reported. In contrast, assessing the integration of physical activity counselling and behaviour change support into routine primary healthcare services proved challenging due to limited data availability. The most recent information, dating back to 2010, indicated that only Belarus, Kazakhstan, and Tajikistan had government-approved national guidelines or protocols in place. No updated data were available for any country, preventing assessment of whether this gap persists or whether additional countries have since adopted such measures. This absence of recent reporting suggests either that implementation has remained limited or that monitoring systems for these interventions are insufficiently developed across the region.

Similarly, our findings are consistent with earlier research highlighting the fragmented and uneven implementation of healthy nutrition policies across the EECA (WHO 2022a). While some progress has been made, particularly in salt reduction strategies and public food procurement policies, many impactful measures remain underutilized or are implemented on a voluntary basis. These include mandatory reformulation to reduce saturated and *trans* fats, front-of-pack nutrition labelling, and regulation of food marketing to children. The absence of sugar-sweetened beverage taxation and varying levels of alignment with the International Code of Marketing of Breast-milk Substitutes further underscore the persistent policy gaps. Despite growing awareness of the health burden linked to poor nutrition, EECA countries continue to face challenges in implementing comprehensive and enforceable policy frameworks.

### Strengths and limitations

This study has several strengths. First, it draws on multiple WHO data sources, providing a coherent and comprehensive overview of NCD policy implementation in the EECA subregion. This assessment relied intentionally on WHO global and regional publications because they provide the authoritative, standardized, and most up-to-date information for evaluating national NCD policy implementation. Second, the use of WHO’s Best Buys framework provides a standardized approach for analysing policy adoption. Third, by examining policy changes over time, the study captures shifts in government priorities and highlights both progress and ongoing challenges. The shared historical context of the EECA countries—many of which had similar policy structures prior to the dissolution of the Soviet Union—offers a unique opportunity to compare divergent NCD policy trajectories.

Our study also has several limitations. It focused on WHO Best Buys, which prioritize cost-effective, population-level interventions but may not encompass all relevant evidence-based policies. For example, interventions targeting youth tobacco use—such as school-based prevention programmes (Thomas et al. 2013), flavour bans (Farley and Johns 2017), or measures addressing emerging tobacco products (Feliu et al. 2023)—are not fully captured within the current Best Buys framework. Further, the study did not aim to conduct a systematic literature review; rather, the objective was to assess country progress against WHO’s normative guidance using indicators that are applied consistently across 12 EECA countries. Reliance on WHO materials ensured methodological comparability, harmonized definitions, and alignment with the official WHO approach to tracking population-level NCD policies. While broader policy implementation frameworks (Erasmus et al. 2014) and various quantitative tools (Allen et al. 2020) exist, these were not applied here because the study did not aim to examine determinants of implementation, but to provide a descriptive, cross-country assessment of policy adoption. Applying full policy-process frameworks—such as those developed by Goyal and Howlett (2019)—would require detailed information on policy stages, settings, stakeholders, and institutional processes that is not available in the global WHO datasets used in this analysis. Implementing such frameworks across 12 countries would necessitate extensive primary data collection in each setting, which lies beyond the scope of this comparative assessment. Moreover, the retrospective reconstruction of such detailed policy process information would be methodologically challenging and subject to significant limitations in data availability and reliability. Additionally, the analysis was constrained by the availability and quality of country-level data. Country Capacity Surveys rely on inputs from Ministries of Health, which may be incomplete due to competing priorities or data gaps. While policy adoption is well-documented, data on actual implementation and enforcement are inconsistent, limiting our ability to assess real-world impact.

### Implications for further research

This study provides the first comprehensive analysis of NCD policy implementation in the EECA subregion, using the WHO NCD Best Buys framework. It offers a valuable tool for guiding future policy development and identifying areas where further action is needed to improve NCD outcomes.

Future research should focus on evaluating the effectiveness of NCD policies in EECA, particularly their implementation, enforcement, and population-level impact. Comparative studies assessing the long-term outcomes of different policy approaches could offer insights into best practices for reducing NCD risk factors. Research should also explore barriers to policy enforcement, including governance structures, financing mechanisms, and multisectoral collaboration.

Given the limited integration of the NCD agenda into emergency preparedness plans, further studies should examine crisis response frameworks in humanitarian settings that address NCDs. Real-world implementation of Best Buy interventions—particularly in resource-limited settings—should be assessed, and factors contributing to successful adoption identified. Strengthening global monitoring frameworks to better align targets with Best Buys and Quick Buy strategies, alongside enhancing data collection and surveillance capacity, will be essential for tracking disease burden, evaluating health system responses, and ensuring equitable access to NCD prevention and treatment across the EECA subregion.

## Conclusions

Despite progress in NCD prevention and control across Eastern Europe and Central Asia, substantial gaps in policy implementation and access to essential services persist. Strengthening tobacco and alcohol control—through higher taxation, comprehensive advertising bans, and improved enforcement—is critical. Evidence-based nutrition policies, such as mandatory *trans*-fat elimination, front-of-pack labelling, restrictions on unhealthy food marketing, and fiscal measures require further prioritization. Similarly, integrating physical activity promotion into health care and community settings remains an important yet undeveloped area.

All countries in the EECA subregion have substantial room to improve their implementation of WHO’s Best Buys to accelerate progress towards achieving SDG 3.4.1, which aims to reduce premature mortality from NCDs. Our findings highlight clear, actionable policy levers that governments can adopt to close these gaps, providing direction for immediate policy strengthening while also identifying areas where data are lacking and thus national assessments may be needed. Given the ongoing challenges and disparities in NCD outcomes, EECA countries require targeted attention and support from global and regional stakeholders to accelerate progress, and achieve long-term health improvements. Policymakers can use these results to prioritize high-impact, cost-effective interventions, strengthen enforcement mechanisms, and align national strategies with WHO recommendations. Conducting in-depth, country-specific analyses on the implementation of WHO’s Best Buys and Quick Buys could provide valuable insights to inform and support more tailored, comprehensive approaches. Such efforts would help ensure that interventions effectively address multiple NCD risk factors, thereby preventing several NCDs driven by these shared risks, and contributing to more equitable and sustainable health outcomes across the subregion.

## Supplementary Material

czag055_Supplementary_Data

## Data Availability

This study uses secondary data from publicly available WHO datasets. The primary source was the WHO NCD Country Capacity Surveys (2010–2023), accessible through the WHO Regional Office for Europe. These were supplemented with publicly available data from the WHO Global Health Observatory, WHO Reports on the Global Tobacco Epidemic, WHO Global Status Reports on Alcohol and Health, WHO NCD Progress Monitor Reports, the WHO NCD Document Repository, and the WHO Status Reports on the Marketing of Breast‑Milk Substitutes. All data sources are cited in the manuscript, and details on which datasets were used for each policy assessment are provided in Appendix A. No proprietary or restricted data were used.

## Reflexivity statement

The authors acknowledge that their professional backgrounds in epidemiology, global health, and noncommunicable diseases (NCDs) have shaped the framing of this study. Their experience in data analysis, health systems, and multicountry research informed both the selection of methods and the interpretation of findings. While these perspectives provided valuable technical expertise, the authors remained attentive to potential biases in assessing progress across diverse country contexts. To mitigate such biases, the analysis was conducted collaboratively, bringing together a research team with diverse expertise and grounding the work in publicly available data sources. The authors recognize that their positions as researchers and experts in epidemiology and NCDs may influence how results are communicated, and they have sought to present the findings in a manner that highlights countries’ progress in implementing effective NCD interventions and amplifies the voices of those most affected by NCDs.
