# Supplementary material for: Assessing the progress in implementing population-based policies to reduce the burden of noncommunicable diseases in Eastern Europe and Central Asia, 2010–2024
Source: Health Policy Plan. 2026 Apr 16;41(6):942–54. doi: 10.1093/heapol/czag055 (PMC13276267; doi:10.1093/heapol/czag055)
Supplement: czag055_Supplementary_Data [file czag055_supplementary_data.zip › Appendix A.docx]

**Appendix A. List, definitions and sources of NCD Best Buys policies assessed**

This appendix contains a table summarizing all WHO NCD “Best Buys” policies evaluated in the study. Each row begins with a **Policy code**, where the initial letter indicates the policy domain – T for tobacco, A for alcohol, H for healthy nutrition, and P for physical activity – followed by the number of the intervention as presented in the WHO “Best Buys” list; subsets assessed separately are further distinguished by decimal numbers (e.g., 1.1, 1.2). The **Cost-effectiveness category** classifies each intervention as *Best* (≤ I$100 per healthy life year [HLY] gained in low- and lower-middle-income countries), *Good* (> I$100 per HLY gained), or *Other* (WHO-recommended interventions without cost-effectiveness estimates). The **Intervention name** column cites the official title of the measure from the 2024 WHO “Best Buys” guide. **Data source** specifies the earliest and latest years of the data used for the assessment, while the **Indicator definition** details how each policy’s implementation was measured. Finally, the **Assigned value** column provides a score from 0 to 1, applied only to cost-effective interventions available for the assessment, to indicate the degree of implementation.

| **Policy code** | **Cost-effective-ness analysis** | **Name of interventions from WHO NCD "Best Buys" guide (2024)** | **Data source (earliest year, latest year assessed)** | **Definition of indicator** | **Assigned value from 0 to 1 (for cost-effective interventions only)** |
| --- | --- | --- | --- | --- | --- |
| **I. Tobacco policies** | | | | | |
| T1 | Best | Increase excise taxes and prices on tobacco products | WHO Health Observatory (2008, 2022) | Country-provided information on taxes and prices is assessed to yield indicators to describe the comparative level of taxes on tobacco products in countries.   Taxes assessed include excise tax, value added tax (“VAT”), import duty (when the cigarettes were imported) and any other taxes levied. Only the price of the most popular brand of cigarettes is considered. In the case of countries where different levels of taxes applied to cigarettes are based on either length, quantity produced or type (e.g. filter vs. non-filter), only the rate that applied to the most popular brand is used in the calculation. Given the lack of information on country and brand-specific profit margins of retailers and wholesalers, their profits were assumed to be zero (unless provided by the national data collector).  The implementation status of the Raise tobacco taxes measure was classified by grouping countries into five groups. The groupings for this indicator are: 1 = Data not reported; 2 = ≤ 25% of retail price is tax; 3 = 26–50% of retail price is tax; 4 = 51–75% of retail price is tax; 5 = >75% of retail price is tax. | 0 = not implemented 0.25 = ≤ 25% of retail price is tax  0.5 = 26–50% of retail price is tax  0.75 = 51–75% of retail price is tax 1.0 = >75% of retail price is tax |
| T2 | Best | Implement large graphic health warnings on all tobacco packages, accompanied by plain/standardized packaging | WHO Health Observatory (2007, 2022) | Country's legislation is assessed to determine whether health warnings with specific criteria are mandated. The groupings for this indicator are: 1 = Data not reported; 2 = No warning or warning covering <30% of pack surface;3 = ≥30% of the front and back of the cigarette pack but no pictures or pictograms and/or *other appropriate characteristics***; 4 = 31%–49% of the front and back of the cigarette pack including pictures or pictograms and other appropriate characteristics**; 5 = ≥50% of the front and back of the cigarette pack including pictures or pictograms and appropriate characteristics**.  *Other appropriate characteristics*** Specific health warnings mandated; appearing on individual packages as well as on any outside packaging and labelling used in retail sale; describing specific harmful effects of tobacco use on health; are large, clear, visible and legible (e.g. specific colors and font style and sizes are mandated); rotate; written in (all) principal language(s) of the country | 0 = data not reported, or warning covering <30% of pack surface 0.33 = ≥30%* but no pictures or pictograms and/or other appropriate characteristics 0.67 = 31%–49%* including pictures or pictograms and other appropriate characteristics 1.0 = ≥50%* including pictures or pictograms and appropriate characteristics |
| T3 | Best | Enact and enforce comprehensive bans on tobacco advertising, promotion and sponsorship | WHO Health Observatory (2007, 2022) | Country's legislation is assessed to determine whether all or any forms of tobacco advertising promotion and sponsorship are banned. The groupings for this indicator are: 1 = Data not reported; 2 = Complete absence of ban, or ban that does not cover national television (TV), radio and print media; 3 = Ban on national TV, radio and print media only; 4 = Ban on national TV, radio and print media as well as on some but not all other forms of *direct** and/or *indirect*** *advertising*; 5 = Ban on all forms of *direct** and *indirect*** advertising.  * *Direct advertising bans*: national television and radio; local magazines and newspapers; billboards and outdoor advertising; point of sale.  *** Indirect advertising bans*: free distribution of tobacco products in the mail or through other means; promotional discounts; non-tobacco products identified with tobacco brand names (brand extension); brand names of non-tobacco products used for tobacco products; appearance of tobacco products in television and/or films; sponsored events. | 0 = data not reported, or warning covering <30% of pack surface, or Complete absence of ban, or ban that does not cover national television (TV), radio and print media; 0.33 = Ban on national TV, radio and print media only; 0.67 = Ban on national TV, radio and print media as well as on some but not all other forms of *direct** and/or *indirect*** *advertising;* 1.0 = Ban on all forms of *direct** and *indirect*** *advertising* |
| T4 | Best | Eliminate exposure to second-hand tobacco smoke in all indoor workplaces, public places and public transport | WHO Health Observatory (2007, 2022) | Country's legislation is assessed to determine whether smoke-free laws exist in each of the following places at either the national or subnational level: health-care facilities; educational facilities other than universities; universities; government facilities; indoor offices; restaurants; pubs and bars; public transport. The implementation status of the Smoke-free environments measure was classified by grouping countries into five groups. The groupings for this indicator are: 1 = Data not reported/not categorized*; 2 = Up to two public places completely smoke-free; 3 = Three to five public places completely smoke-free; 4 = Six to seven public places completely smoke-free; 6 = All public places completely smoke-free (or at least 90% of the population covered by complete subnational smoke-free legislation)  *In several countries, in order to significantly expand the creation of smoke-free places, including restaurants and bars, it was politically necessary to include exceptions to the law that allowed for the provision of designated smoking rooms. The requirements for designated smoking rooms are technically so complex and stringent that, for practical purposes, few or no establishments are expected to implement them. Because no data were requested on the number of complex designated smoking rooms actually constructed, it is not possible to know whether these laws have resulted in the complete absence of such rooms as intended. For this reason, these few countries have not been categorized in the analyses for this indicator. | 0 = Data not reported/not categorized 0.25 = Up to two public places completely smoke-free  0.5 = Three to five public places completely smoke-free  0.75 = Six to seven public places completely smoke-free  1.0 = All public places completely smoke-free (or at least 90% of the population covered by complete subnational smoke-free legislation) |
| T5 | Best | Implement effective mass media campaigns that educate the public about the harms of smoking/tobacco use and secondhand smoke, and encourage behaviour change | WHO Health Observatory (2010, 2022) | The implementation status of the Anti-tobacco mass media measure was classified by grouping countries into five groups. The groups for this indicator are: 1 = Data not reported; 2 = No national campaign conducted in the reporting period with a duration of at least three weeks; 3 = National campaign conducted with 1-4 appropriate *characteristics**; 4 = National campaign conducted with 5-6 appropriate *characteristics**, or with 7 *characteristics* excluding airing on TV and/or radio; 5 = National campaign conducted with at least 7 appropriate *characteristics** including airing on TV and/or radio.  **Characteristics* of a high-quality campaign are: the campaign was part of a tobacco control programme; before the campaign, research was undertaken or reviewed to gain a thorough understanding of the target audience; campaign communications materials were pretested with the target audience and refined in line with campaign objectives; air time (radio, television) and/or placement (billboards, print advertising, etc.) was obtained by purchasing or securing it using either the organization’s own internal resources or an external media planner or agency (this information indicates whether the campaign adopted a thorough media planning and buying process to effectively and efficiently reach its target audience); the implementing agency worked with journalists to gain publicity or news coverage for the campaign; process evaluation was undertaken to assess how effectively the campaign had been implemented; and an outcome evaluation process was implemented to assess the campaign impact. | 0 = Data not reported or No national campaign conducted in the reporting period with a duration of at least three weeks 0.33 = National campaign conducted with 1-4 appropriate characteristics* 0.67 = National campaign conducted with 5-6 appropriate characteristics*, or with 7 characteristics excluding airing on TV and/or radio 1.0 = National campaign conducted with at least 7 appropriate characteristics* including airing on TV and/or radio. |
| T6 | Good | Provision of cost-covered effective population-wide support (including brief advice, national toll-free quit line services and mCessation) for tobacco cessation to all tobacco users | WHO Health Observatory (2007, 2022) | Includes both T6&T7 Information from countries on the availability and non-availability of particular tobacco cessation aids is assessed to determine the comparative level of assistance countries provide to help tobacco users quit. The groupings for this indicator are: 1 = Data not reported; 2 = None; 3 = nicotine replacement therapy and/or some *cessation services*** (neither cost-covered); 4 = nicotine replacement therapy and/or some *cessation services*** (at least one of which is cost-covered); 5 = National quit line, and both nicotine replacement therapy and some *cessation services*** cost-covered.  *** Smoking cessation support* available in any of the following places: health clinics or other primary care facilities, hospitals, office of a health professional, the community. | 0 = Data not reported, or not available 0.33 = NRT* and/or some cessation services** (neither cost-covered)  0.67 = NRT* and/or some cessation services** (at least one of which is cost-covered) 1.0 = National quit line, and both NRT* and some cessation services** cost-covered * |
| T7 | Good | Provision of cost-covered effective pharmacological interventions to all tobacco users who want to quit, through the use of nicotine replacement therapy (NRT), Bupropion and Varenicline. |  |  |  |
| T8 | Other | Establish a tracking and tracing system to support the elimination of illicit trade in tobacco products that is in line with Article 8 of the Protocol to Eliminate Illicit Trade in Tobacco Products | Data source not found; not assessed | Data source not found; not assessed | Data source not found; not assessed |
| T9 | Other | Ban cross-border tobacco advertising, promotion and sponsorship, including those through modern means of communication | Data source not found; not assessed | Data source not found; not assessed | Data source not found; not assessed |
| **II. Alcohol policies** | | | | | |
| A1 | Best | Increase excise taxes on alcoholic beverages | NCD progress monitor reports (2015, 2022) | ○ = not achieved ◐ = partially achieved ● = fully achieved, − = documentation not available, DK = don't know | 0 = increase in excise taxes on alcoholic beverages not implemented, no data, or don't know  0.5 = increase in excise taxes on alcoholic beverages is partially achieved 1.0 = increase in excise taxes on alcoholic beverages is fully achieved |
| A2 | Best | Enact and enforce bans or comprehensive restrictions on exposure to alcohol advertising (across multiple types of media) | NCD progress monitor reports (2015, 2022) | ○ = not achieved ◐ = partially achieved ● = fully achieved, − = documentation not available, DK = don't know | 0 = bans or comprehensive restrictions on exposure to alcohol advertising not achieved, no data, or don't know 0.5 = partially achieved 1.0 = fully achieved |
| A3 | Best | Enact and enforce restrictions on the physical availability of retailed alcohol (via reduced hours of sale) | NCD progress monitor reports (2015, 2022) | ○ = not achieved ◐ = partially achieved ● = fully achieved, − = documentation not available, DK = don't know | 0 = restrictions on the physical availability of retailed alcohol not achieved, no data, or don't know 0.5 = restrictions on the physical availability of retailed alcohol is partially achieved 1.0 = restrictions on the physical availability of retailed alcohol is fully achieved |
| A4 | Good | Enact and enforce drink-driving laws and blood alcohol concentration limits via sobriety checkpoints | Global status report on alcohol (2014, 2018) | Value of blood alcohol concentration limits, if given | 0 = concentration limits are not indicated  1.0 = concentration limits are indicated |
| A5 | Good | Provide brief psychosocial intervention for persons with hazardous and harmful alcohol use | Global status report on alcohol (2014, 2018) | Screening and brief intervention programmes for alcohol: 4- Available, 3- Limited availability, 2-Missing data, 1-Not available | 0 = brief psychosocial intervention for persons with hazardous and harmful alcohol use are not available or missing data 0.5 = limited availability of brief psychosocial intervention for persons with hazardous and harmful alcohol use  1.0 = brief psychosocial intervention for persons with hazardous and harmful alcohol use is available |
| A6 | Other | Carry out regular reviews of prices in relation to levels of inflation and income | Data source not found; not assessed | Data source not found; not assessed | Data source not found; not assessed |
| A7 | Other | Establish minimum prices for alcohol where applicable | Data source not found; not assessed | Data source not found; not assessed | Data source not found; not assessed |
| A8 | Other | Enact and enforce an appropriate minimum age for purchase or consumption of alcoholic beverages and reduce density of retail outlets | Data source not found; not assessed | Data source not found; not assessed | Data source not found; not assessed |
| A9 | Other | Restrict or ban promotions of alcoholic beverages in connection with sponsorships and activities targeting young people | Data source not found; not assessed | Data source not found; not assessed | Data source not found; not assessed |
| A10 | Other | Provide prevention, treatment and care for alcohol use disorders and comorbid conditions in health and social services | Global status report on alcohol (-, 2024) | Service Capacity Index for Alcohol Use Disorders (SCI-AUD) (Direct, Imputation, Missing data, Not included in estimation) | Not applicable |
| A11 | Other | Provide consumers with information, including labels and health warnings, about content of alcoholic beverages and the harms associated with alcohol consumption | Global status report on alcohol (-, 2024) | Health warning labels regarding pregnancy, underage drinking, drink-driving and cancer on advertisements and containers; consumer information displayed on containers, number of standard alcoholic drinks displayed on containers, alcohol content displayed on containers | Not applicable |
| **III. Unhealthy diet** | | | | | |
| H1 | Best | Reformulation policies for healthier food and beverage products (e.g. elimination of trans-fatty acids and/or reduction of saturated fats, free sugars and/or sodium) | Assessed separately for saturated fats, trans-fatty acids and sodium; information on reformulation policies for free sugars not found | | |
| H1.1 | Best | Reformulation policies for reduction of saturated fats | Country Capacity Survey (2015, 2023) | Country introduced reformulation policies on the reduction of saturated fats intake: 0 - not reported, no or don’t know, 1 - yes and  If yes, are these policies 1= voluntary/ self-regulating, 2 = mandatory/government legislation, 0 = don't know | 0 = reformulation policies on the reduction of saturated fats intake, not implemented, no data or don't know  0.5 = yes, voluntary/self-regulating OR yes, reformulation policies on the reduction of saturated fats implemented but don't know whether they are voluntary or mandatory 1.0 = yes, mandatory/government legislation reformulation policies on the reduction of saturated fats |
| H1.2 | Best | Reformulation policies for trans-fatty acids | Country Capacity Survey (2019, 2023) | Country introduced reformulation policies on the reduction of trans-fatty acids 0 - not reported, no or don’t know, 1 - yes and  If yes (2019) are this policies 1= voluntary/ self-regulating, 2 = mandatory/government legislation, 0 (77) = don't know If yes (2023) are these policies 1= Mandatory national ban on the production or use of partially hydrogenated oils (PHO) as an ingredient in all foods or Mandatory national limit of 2g of industrially produced trans-fatty acids per 100g of total fat in all foods, 0 (77) = don't know | 0 = reformulation policies on the reduction of trans-fatty acids not implemented, no data or don't know  0.5 = yes, voluntary/self-regulating OR yes, reformulation policies on the reduction of trans-fatty acids implemented but don't know whether they are voluntary or mandatory 1.0 = yes, mandatory/government legislation or yes, mandatory national ban on the production or use of partially hydrogenated oils (PHO) as an ingredient in all foods or, yes, mandatory national limit of 2g of industrially produced trans-fatty acids per 100g of total fat in all foods |
| H1.3 | Best | Reformulation policies for sodium | Country Capacity Survey (2015, 2023) | Country introduced reformulation policies on the reduction of salt/sodium: 0 - not reported, no or don’t know, 1 - yes | 0 = reformulation policies on the reduction of salt/sodium not implemented, no data or don't know  1.0 = yes, reformulation policies on the reduction of salt/sodium implemented |
| H2 | Best | Front-of-pack labelling as part of comprehensive nutrition labelling policies for facilitating consumers’ understanding and choice of food for healthy diets | Country Capacity Survey (2021, 2023) | Implementation of the front-of-pack labelling as part of comprehensive nutrition labelling policies for facilitating consumers’ understanding and choice of food for healthy diets (yes, no, don't know) | 0 = front-of-pack labelling not implemented, no data, do not know 1 = front-of-pack labelling policies implemented |
| H3 | Best | Public food procurement and service policies for healthy diets (e.g. to reduce the intake of free sugars, sodium and unhealthy fats, and to increase the consumption of legumes, wholegrains, fruits and vegetables) | Country Capacity Survey (-, 2023) | Implementation of the food procurement and service policies for various settings (e.g. schools, government offices, public events) to reduce the content of saturated fatty acids, trans-fatty acids, sugars or salt/sodium in food served or sold (yes, no, don't know) | 0 = food procurement policies and service policies for various settings (e.g. schools, government offices, public events) to reduce the content of saturated fatty acids, trans-fatty acids, sugars or salt/sodium in food served or sold not implemented, no data or don't know 1 = food procurement policies and service policies for various settings (e.g. schools, government offices, public events) to reduce the content of saturated fatty acids, trans-fatty acids, sugars or salt/sodium in food served or sold implemented |
| H4 | Best | Behaviour change communication and mass media campaign for healthy diets (e.g. to reduce the intake of energy, free sugars, sodium and unhealthy fats, and to increase the consumption of legumes, wholegrains, fruits and vegetables) | Country Capacity Survey (2015, 2023) | 2015: implemented any national public awareness programme on diet within the past 5 years (yes, no, don't know) 2023: implemented any national public awareness programme on diet within the past 2 years (yes, no, don't know) | 0 = Behaviour change communication and mass media campaign for healthy diets not implemented, no data or don't know 1 = Behaviour change communication and mass media campaign for healthy diets implemented |
| H5 | Best | Policies to protect children from the harmful impact of food marketing on diet | Country Capacity Survey (2015, 2023) | Countries implement policies to protect children from the harmful impact of food marketing on diet (yes, know, don't know) | 0 = policies to protect children from the harmful impact of food marketing on diet not implemented, no data or don't know 1.0 = yes, policies to protect children from the harmful impact of food marketing on diet implemented |
| H6 | Best | Protection, promotion and support of optimal breastfeeding practices | Marketing of breast-milk substitutes: national implementation of the international code, status report (2016, 2024) | 3= Full provisions in law (before 2020) or Substantially aligned with the Code (since 2020): countries have enacted legislation or adopted regulations, decrees or other legally binding measures encompassing all or nearly all provisions of the Code and subsequent WHA resolutions; 2= Many provisions in law: countries have enacted legislation or adopted regulations, decrees or other legally binding measures encompassing many provisions of the Code and subsequent WHA resolutions; 1= Few provisions in law: countries have enacted legislation or adopted regulations, directives, decrees or other legally binding measures covering few of the provisions of the Code or subsequent WHA resolutions; 0 = No legal measures: countries have taken no action or have implemented the Code only through voluntary agreements or other non-legal measures (includes countries that have drafted legislation but not enacted it); 0 = No information: countries for which WHO, UNICEF, and IBFAN/ICDC have been unable to obtain information on the legal status of the Code. | 0 = no information or no legal measures: countries have taken no action or have implemented the Code only through voluntary agreements or other non-legal measures (includes countries that have drafted legislation but not enacted it);  0.33 = Few provisions in law: countries have enacted legislation or adopted regulations, directives, decrees or other legally binding measures covering few of the provisions of the Code or subsequent WHA resolutions 0.67= Many provisions in law: countries have enacted legislation or adopted regulations, decrees or other legally binding measures encompassing many provisions of the Code and subsequent WHA resolutions 1.0 = Substantially aligned with the Code/Full provisions in law |
| H7 | Good | Taxation on sugar-sweetened beverages as part of comprehensive fiscal policies to promote healthy diets | Country Capacity Survey (2015, 2023) | Country implements taxation on sugar-sweetened beverages as part of comprehensive fiscal policies to promote healthy diets (yes, no, don't know) | 0 = no taxation policies on sugar-sweetened beverages, no data or don't know 1 = yes, taxation on sugar-sweetened beverages implemented |
| H8 | Other | Subsidies on healthy foods and beverages (e.g. fruits and vegetables) as part of fiscal policies for healthy diets | Country Capacity Survey (2015, 2023) | Country provides price subsidies for healthy foods (yes, no, don't know) | Not applicable |
| H9 | Other | Menu labelling in food service for healthy diets (e.g. to reduce the intake of energy, free sugars, sodium and/or unhealthy fats): B24d (H2_1.11) Other interpretive labelling (e.g. menu or vending machine labelling, back of pack warning messages) | Country Capacity Survey (- , 2023) | Other interpretive labelling (e.g. menu or vending machine labelling, or back of pack warning messages) (yes, no, don't know) | Not applicable |
| H10 | Other | Limiting portion and package size for healthy diet (e.g. to reduce the intake of energy, free sugars, sodium and/or unhealthy fats) | Data source not found; not assessed | Data source not found; not assessed | Data source not found; not assessed |
| H11 | Other | Nutrition education and counselling for healthy diets in different settings (e.g. in preschools, schools, workplaces and hospitals) | Country Capacity Survey (2010, -) | Country has recognized / government approved evidence-based national guidelines/protocols/standards for dietary counseling (yes, no, don't know); if yes, are they being implemented? | Not applicable |
| **IV. Physical Activity** | | | | | |
| P1 | Best | Implement sustained, population wide, best practice communication campaigns to promote physical activity, with links to community-based programmes and environmental improvements to enable and support behaviour change. | Country Capacity Survey (2015, 2023) | 2015: Country implemented any national public awareness programme on physical activity within the past 5 years (yes, no, don't know) 2023: Country implemented any national public awareness programme on physical activity within the past 2 years (yes, no, don't know) | 0 = no physical activity campaign implemented, no data or don't know 1 = physical activity campaign implemented |
| P2 | Good | Provide physical activity assessment, counselling, and behaviour change support as part of routine primary health care services through the use of a brief intervention | Country Capacity Survey (2010, -) | The country has recognized / government approved evidence-based national guidelines/protocols/standards for physical activity counseling (yes, no, don't know) | 0 = no recognized /government approved evidence-based national guidelines/protocols/standards for physical activity counseling, no data or don't know 1 = yes, the country has recognized /government approved evidence-based national guidelines/protocols/standards for physical activity counseling |
| P3 | Other | Implement urban and transport planning and urban design, at all levels of government, to provide compact neighbourhoods providing mixed-land use and connected networks for walking | Data source not found; not assessed | Data source not found; not assessed | Data source not found; not assessed |
| P4 | Other | Implement whole-of-school programmes that include quality physical education, and adequate facilities, equipment and programs supporting active travel to/from school and support physical activity for all children of all abilities during and after school | Data source not found; not assessed | Data source not found; not assessed | Data source not found; not assessed |
| P5 | Other | Improve walking and cycling infrastructure ensuring universal and equitable access to enable and promote safe walking, cycling, other forms of micro mobility (e.g. wheelchairs, scooters and skates) by people of all ages and abilities | Data source not found; not assessed | Data source not found; not assessed | Data source not found; not assessed |
| P6 | Other | Implement multi-component workplace physical activity programmes | Data source not found; not assessed | Data source not found; not assessed | Data source not found; not assessed |
| P7 | Other | Provide and promote physical activity through provision of community-based (grass roots) sport and recreation programmes and conduct free mass participation events to encourage engagement by people of all ages and abilities | Data source not found; not assessed | Data source not found; not assessed | Data source not found; not assessed |
