# Supplementary material for: Assessing the progress in implementing population-based policies to reduce the burden of noncommunicable diseases in Eastern Europe and Central Asia, 2010–2024
Source: Health Policy Plan. 2026 Apr 16;41(6):942–54. doi: 10.1093/heapol/czag055 (PMC13276267; doi:10.1093/heapol/czag055)
Supplement: czag055_Supplementary_Data [file czag055_supplementary_data.zip › Appendix B.docx]

**Appendix B. Assigned values to policies on tobacco, alcohol, physical activity and healthy nutrition across twelve countries of Eastern Europe and Central Asia**

**Table B.1 Assigned values for tobacco policies (earliest year, latest year): T1** - Increase excise taxes and prices on tobacco products (2008, 2022); **T2** - Implement large graphic health warnings on all tobacco packages, accompanied by plain/standardized packaging (2007, 2022); **T3** - Enact and enforce comprehensive bans on tobacco advertising, promotion and sponsorship (2007, 2022); **T4** - Eliminate exposure to second-hand tobacco smoke in all indoor workplaces, public places and public transport (2007, 2022); **T5** - Implement effective mass media campaigns that educate the public about the harms of smoking/tobacco use and second-hand smoke, and encourage behaviour change (2010, 2022); **T6** – Provide cost-covered, effective, population-wide support (including brief advice, national toll-free quit line services and mCessation) for tobacco cessation to all tobacco users, and provide cost-covered effective pharmacological interventions to all tobacco users who want to quit, through the use of nicotine replacement therapy, bupropion and varenicline (2007, 2022).

| **Country** | **T1** | | **T2** | | **T3** | | **T4** | | **T5** | | **T6** | |
| --- | --- | --- | --- | --- | --- | --- | --- | --- | --- | --- | --- | --- |
|  | **Earliest** | **Latest** | **Earliest** | **Latest** | **Earliest** | **Latest** | **Earliest** | **Latest** | **Earliest** | **Latest** | **Earliest** | **Latest** |
| Armenia | 0.25 | 0.50 | 0.33 | 1.00 | 0.00 | 1.00 | 0.50 | 0.75 | 0.00 | 0.33 | 0.33 | 0.67 |
| Azerbaijan | 0.25 | 0.75 | 0.00 | 0.33 | 0.67 | 1.00 | 0.50 | 0.50 | 0.00 | 0.67 | 0.00 | 0.33 |
| Belarus | 0.50 | 0.75 | 0.00 | 1.00 | 0.67 | 0.67 | 0.25 | 0.25 | 0.00 | 0.67 | 0.67 | 0.67 |
| Georgia | 0.50 | 0.75 | 0.33 | 1.00 | 0.00 | 0.67 | 0.25 | 0.75 | 0.67 | 0.67 | 0.33 | 0.33 |
| Kazakhstan | 0.25 | 0.75 | 0.33 | 1.00 | 0.67 | 0.67 | 0.50 | 0.75 | 1.00 | 0.67 | 0.67 | 0.67 |
| Kyrgyzstan | 0.50 | 0.75 | 0.33 | 1.00 | 0.00 | 1.00 | 0.50 | 1.00 | 0.00 | 0.00 | 0.33 | 0.67 |
| Moldova | 0.25 | 0.75 | 0.33 | 1.00 | 0.67 | 1.00 | 0.50 | 1.00 | 0.33 | 0.00 | 0.33 | 0.67 |
| Russia | 0.50 | 0.75 | 0.00 | 1.00 | 0.00 | 1.00 | 0.25 | 1.00 | 1.00 | 1.00 | 0.33 | 0.67 |
| Tajikistan | 0.00 | 0.75 | 0.00 | 1.00 | 0.67 | 0.67 | 0.25 | 1.00 | 0.00 | 0.00 | 0.33 | 0.00 |
| Turkmenistan | 0.50 | 0.50 | 0.00 | 1.00 | 0.00 | 0.67 | 1.00 | 1.00 | 0.00 | 1.00 | 0.67 | 0.67 |
| Ukraine | 0.50 | 0.75 | 0.33 | 1.00 | 0.00 | 1.00 | 0.25 | 1.00 | 0.00 | 1.00 | 0.33 | 0.33 |
| Uzbekistan | 0.50 | 0.75 | 0.00 | 0.33 | 0.00 | 0.67 | 0.25 | 0.25 | 0.00 | 0.33 | 0.33 | 0.67 |

**Table B.2 Assigned values for alcohol policies (earliest year, latest year): A1** - Increase excise taxes on alcoholic beverages (2015, 2022); **A2** - Enact and enforce bans or comprehensive restrictions on exposure to alcohol advertising (across multiple types of media) (2015, 2022); **A3** - Enact and enforce restrictions on the physical availability of retailed alcohol (via reduced hours of sale) (2015, 2022); **A4** - Enact and enforce drink‑driving laws and blood alcohol concentration limits via sobriety checkpoints (2014, 2018); **A5** - Provide brief psychosocial intervention for persons with hazardous and harmful alcohol use (-, 2024)

| **Country** | **A1** | | **A2** | | **A3** | | **A4** | | **A5** | |
| --- | --- | --- | --- | --- | --- | --- | --- | --- | --- | --- |
|  | **Earliest** | **Latest** | **Earliest** | **Latest** | **Earliest** | **Latest** | **Earliest** | **Latest** | **Earliest** | **Latest** |
| Armenia | 0.50 | 0.50 | 0.50 | 0.50 | 0.50 | 0.50 | 1.00 | 1.00 | 0.00 | 0.00 |
| Azerbaijan | 1.00 | 0.50 | 0.50 | 0.00 | 0.50 | 0.50 | 1.00 | 1.00 | 0.00 | 0.50 |
| Belarus | 1.00 | 1.00 | 0.50 | 1.00 | 0.50 | 0.50 | 1.00 | 1.00 | 0.00 | 1.00 |
| Georgia | 1.00 | 0.50 | 0.50 | 1.00 | 0.50 | 0.50 | 1.00 | 1.00 | 0.00 | 0.50 |
| Kazakhstan | 0.50 | 0.50 | 0.50 | 1.00 | 0.50 | 0.50 | 1.00 | 1.00 | 0.00 | 1.00 |
| Kyrgyzstan | 0.00 | 1.00 | 1.00 | 1.00 | 0.50 | 0.50 | 1.00 | 0.00 | 0.00 | 1.00 |
| Moldova | 0.50 | 0.00 | 0.50 | 0.50 | 0.50 | 0.50 | 0.00 | 1.00 | 0.00 | 0.50 |
| Russia | 1.00 | 0.50 | 1.00 | 1.00 | 0.50 | 0.50 | 0.00 | 1.00 | 0.00 | 1.00 |
| Tajikistan | 1.00 | 0.50 | 0.00 | 0.50 | 0.50 | 0.50 | 1.00 | 1.00 | 0.00 | 1.00 |
| Turkmenistan | 0.50 | 1.00 | 1.00 | 1.00 | 0.50 | 1.00 | 1.00 | 1.00 | 0.00 | 1.00 |
| Ukraine | 0.50 | 0.50 | 0.50 | 1.00 | 0.50 | 0.50 | 1.00 | 1.00 | 0.00 | 0.50 |
| Uzbekistan | 0.50 | 0.50 | 1.00 | 1.00 | 0.50 | 0.50 | 1.00 | 1.00 | 0.00 | 0.00 |

**Table B.3 Assigned values for policies on physical activity (earliest year, latest year): P1** - Implement sustained, population-wide, best-practice communication campaigns to promote physical activity, with links to community-based programmes and environmental improvements to enable and support behaviour change (2015, 2023); **P2** - Provide physical activity assessment, counselling, and behaviour change support as part of routine primary health care services through the use of a brief intervention (2010, -)

|  | **P1** | | **P2** | |
| --- | --- | --- | --- | --- |
| **Country** | **Earliest** | **Latest** | **Earliest** | **Latest** |
| Armenia | 0.00 | 1.00 | 0.00 | 0.00 |
| Azerbaijan | 1.00 | 1.00 | 0.00 | 0.00 |
| Belarus | 1.00 | 1.00 | 0.50 | 0.00 |
| Georgia | 0.00 | 1.00 | 0.00 | 0.00 |
| Kazakhstan | 1.00 | 1.00 | 0.50 | 0.00 |
| Kyrgyzstan | 1.00 | 1.00 | 0.00 | 0.00 |
| Moldova | 0.00 | 1.00 | 0.00 | 0.00 |
| Russia | 0.00 | 1.00 | 0.00 | 0.00 |
| Tajikistan | 1.00 | 0.00 | 0.50 | 0.00 |
| Turkmenistan | 1.00 | 0.00 | 0.00 | 0.00 |
| Ukraine | 0.00 | 0.00 | 0.00 | 0.00 |
| Uzbekistan | 1.00 | 1.00 | 0.00 | 0.00 |

**Table B.4 Assigned values for policies on healthy nutrition (earliest year, latest year): H1** – Reformulation policies for healthier food and beverage products - assessed separately for **H1.1** – reduction of saturated fats (2015, 2023), **H1.2** – elimination of trans-fatty acids (2019, 2023), and **H1.3** – reduction of sodium (2015, 2023); **H2** – Front-of-pack labelling as part of comprehensive nutrition labelling policies for facilitating consumers’ understanding and choice of food for healthy diets (2021, 2023); **H3** – Public food procurement and service policies for healthy diets (-, 2023); **H4** – Behaviour-change communication and mass-media campaigns for healthy diets (2015, 2023); **H5** - Policies to protect children from the harmful impact of food marketing on diet (2015, 2023); **H6** - Protection, promotion, and support of optimal breastfeeding practices (2016, 2024); **H7** - Taxation on sugar-sweetened beverages as part of comprehensive fiscal policies to promote healthy diets (2015, 2023)

| **Country** | **H1.1** | | **H1.2** | | **H1.3** | | **H2** | | **H3** | | **H4** | | **H5** | | **H6** | | **H7** | |
| --- | --- | --- | --- | --- | --- | --- | --- | --- | --- | --- | --- | --- | --- | --- | --- | --- | --- | --- |
|  | **Earliest** | **Latest** | **Earliest** | **Latest** | **Earliest** | **Latest** | **Earliest** | **Latest** | **Earliest** | **Latest** | **Earliest** | **Latest** | **Earliest** | **Latest** | **Earliest** | **Latest** | **Earliest** | **Latest** |
| Armenia | 0.00 | 1.00 | 1.00 | 0.50 | 0.00 | 0.00 | 0.00 | 0.00 | 0.00 | 1.00 | 0.00 | 1.00 | 0.00 | 0.00 | 1.00 | 1.00 | 0.00 | 0.00 |
| Azerbaijan | 0.00 | 0.00 | 1.00 | 0.00 | 0.00 | 0.00 | 0.00 | 0.00 | 0.00 | 1.00 | 1.00 | 1.00 | 0.00 | 0.00 | 0.67 | 0.33 | 0.00 | 0.00 |
| Belarus | 1.00 | 0.00 | 1.00 | 0.50 | 1.00 | 1.00 | 0.00 | 0.00 | 0.00 | 1.00 | 1.00 | 1.00 | 1.00 | 1.00 | 0.00 | 0.00 | 0.00 | 0.00 |
| Georgia | 0.00 | 0.00 | 1.00 | 0.50 | 0.00 | 1.00 | 0.00 | 1.00 | 0.00 | 1.00 | 0.00 | 0.00 | 0.00 | 0.00 | 1.00 | 0.67 | 0.00 | 0.00 |
| Kazakhstan | 0.00 | 0.00 | 1.00 | 0.50 | 1.00 | 1.00 | 0.00 | 0.00 | 0.00 | 1.00 | 1.00 | 1.00 | 0.00 | 0.00 | 0.33 | 0.33 | 0.00 | 0.00 |
| Kyrgyzstan | 1.00 | 1.00 | 1.00 | 0.50 | 1.00 | 0.00 | 0.00 | 0.00 | 0.00 | 1.00 | 1.00 | 1.00 | 1.00 | 0.00 | 0.67 | 0.67 | 0.00 | 0.00 |
| Moldova | 1.00 | 1.00 | 0.50 | 1.00 | 0.00 | 1.00 | 0.00 | 0.00 | 0.00 | 1.00 | 0.00 | 1.00 | 1.00 | 0.00 | 0.33 | 0.33 | 0.00 | 0.00 |
| Russia | 1.00 | 0.50 | 1.00 | 0.50 | 1.00 | 1.00 | 0.00 | 1.00 | 0.00 | 1.00 | 0.00 | 1.00 | 1.00 | 1.00 | 0.33 | 0.33 | 0.00 | 0.00 |
| Tajikistan | 0.50 | 0.00 | 0.00 | 0.00 | 1.00 | 1.00 | 0.00 | 0.00 | 0.00 | 0.00 | 1.00 | 1.00 | 1.00 | 1.00 | 0.67 | 0.67 | 0.00 | 0.00 |
| Turkmenistan | 0.00 | 0.00 | 1.00 | 0.00 | 1.00 | 1.00 | 0.00 | 0.00 | 0.00 | 1.00 | 1.00 | 0.00 | 0.00 | 0.00 | 0.33 | 0.33 | 0.00 | 0.00 |
| Ukraine | 0.00 | 1.00 | 0.00 | 1.00 | 0.00 | 1.00 | 0.00 | 0.00 | 0.00 | 1.00 | 0.00 | 1.00 | 0.00 | 0.00 | 0.33 | 0.33 | 0.00 | 0.00 |
| Uzbekistan | 1.00 | 0.50 | 1.00 | 0.50 | 1.00 | 1.00 | 0.00 | 1.00 | 0.00 | 1.00 | 1.00 | 1.00 | 1.00 | 0.00 | 0.33 | 0.33 | 0.00 | 0.00 |
